# Military Personals

**Published:** 1918-07

**Authors:** 


### MILITARY PERSONALS

Lt. Arthur S. Bugbee of Medina is reported wounded in action.

To Camp Gordon, Atlanta, Ga.; Lieut. Edwin C. Foster, Penn Yan.

To Camp Greene, Charlotte, N. C.; Capt. Edward Dowdle, Oswego.

To Camp Wheeler, Macon, Ga.; Capt. Frederick W. Lester, Seneca Falls.

To Camp Hancock, Augusta, Ga.; Major Isaac W. Brewer, Geneva.

To Camp Meade, Md.; Major Ross Chapman, Binghamton.

To Camp Upton, N. Y.; Lieut. John H. Watson, Buffalo.

To Fort Logan, Col.; Capt. Noah P. Norman, Watkins.

To Fort McHenry, Md.; Lieut. F. P. Schenkelberger, Gowanda.

To Fort Oglethorpe, Ga.; Lieuts. Raymond G. Bell, Auburn; Harold Johnson, Buffalo; G. C. Barone, Lockport; Thomas M. Calladine, Perry; Chas. C. Teresi, Rochester; Albert R. Ellison, Spencer; Urban A. Fischer, Buffalo.

To Fort Wayne, Mich.; Lieut. Leon S. Kurek, Buffalo.

To Rochester, Minn.; Lieut. Roy J. Marshall, Rome.

To Washington, D. C.; Capts. Herman L. Raymond, Collins; Wm. R. Woodbury, Rochester.

To Camp Lee, Va.; Lieut. Ray Woodward, Lackawanna.

To Camp Pike, Ark.; Lieut. Judson J. Wilson, Ithaca.

To Camp Sevier, S. C.; Lieuts. Henry M. Spofford, Batavia; John C. Desloch, Rochester.

To Edgewood, Md.; Capt. Frederick W. Filsinger, Buffalo.

To Camp Jackson, Columbia, S. C.; Carl Tompkins, Buffalo.

To Fort Sam Houston, Texas; Capt. James C. Davis, Rochester.

To Rockefeller Institute; Lieuts. Frederick W. Palmer, Buffalo; Louis S. Kelley, Newark.

